# Circulating extracellular vesicle-encapsulated microRNA as screening biomarkers for intraductal papillary mucinous neoplasm

**DOI:** 10.3892/ol.2021.12872

**Published:** 2021-06-17

**Authors:** Yuki Sato, Rei Suzuki, Tadayuki Takagi, Mitsuru Sugimoto, Hiromasa Ohira

Oncol Lett 20: Article no. 315, 2020; DOI: 10.3892/ol.2020.12178

Subsequently to the publication of the above article, the authors have realized that they incorporated some wrong values for the P-values shown in Fig. 4.

A corrected version of Fig. 4 is shown opposite, featuring all the correct P-values and statistical information. Note that the errors in the Figure did not affect either the results or the conclusions reported in this study. The authors are grateful to the Editor of *Oncology Letters* for granting them the opportunity to publish this corrigendum, and regret any inconvenience caused to the readership of the Journal.

## Figures and Tables

**Figure 4.**
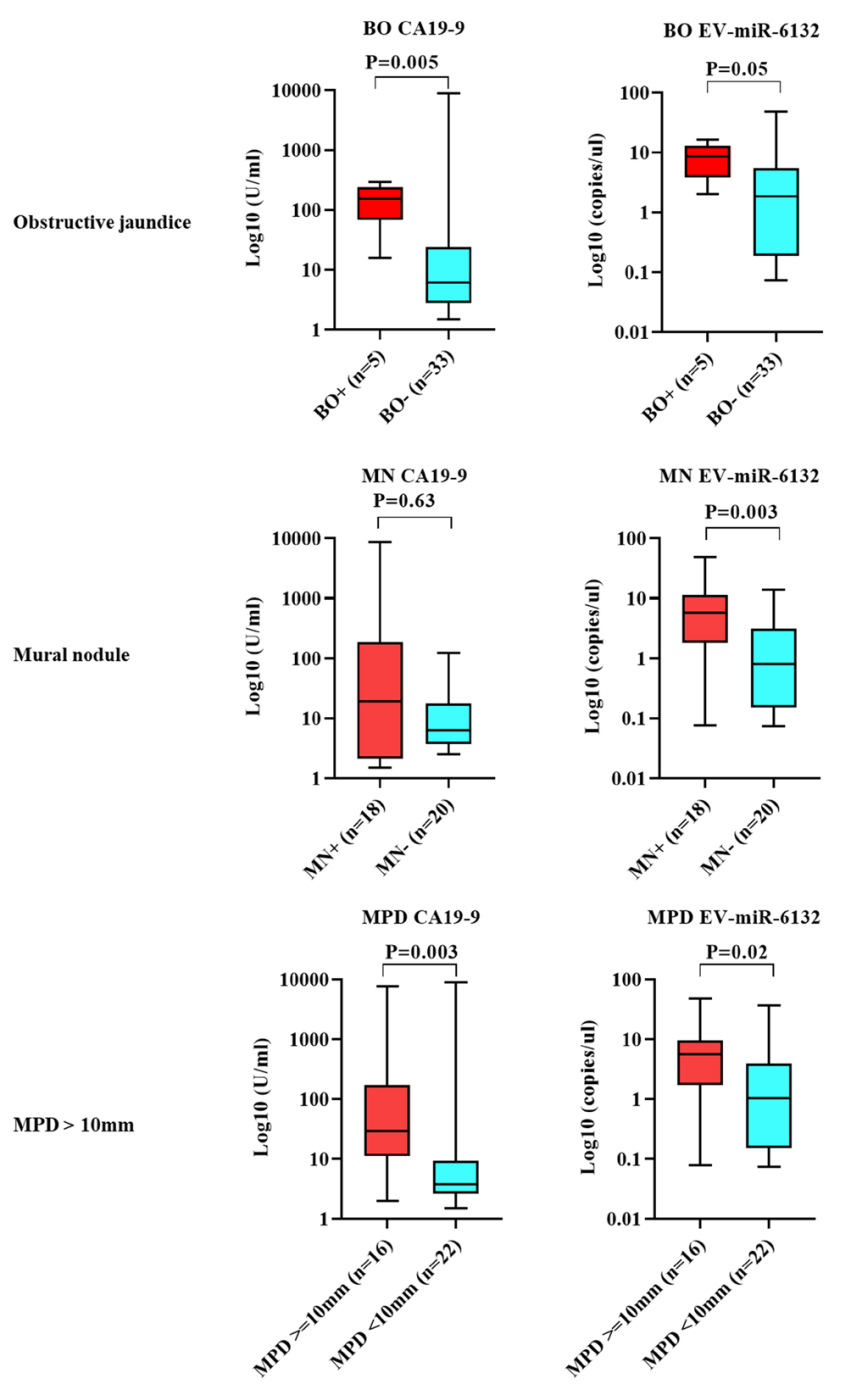
Expression levels of biomarkers per high-risk indicator. CA19-9 was highly expressed in patients with BO (P=0.005). Data were presented in box-and-whisker plot and error bars in figures were presented with range (minimum to maximum). CA19-9, carcinogenic antigen 19-9l EV, extracellular vesicle; miRNA/miR, microRNA; BO, biliary obstruction; MN, mural nodule; MPD, main pancreatic duct.

